# Background Masking Alters Accuracy and Confusion Structure in Mandarin Emotional Prosody Perception

**DOI:** 10.3390/bs16091605

**Published:** 2026-09-09

**Authors:** Yu Dong, Wanyu Hong, Jingyu Zhang, Fuyi Han, Jinghan Zeng

**Affiliations:** 1 Cognitive Science and Allied Health School, Beijing Language and Culture University, Beijing 100083, China; dyxiaoyu0115@blcu.edu.cn (Y.D.); 202421198246@stu.blcu.edu.cn (W.H.); 202421198239@stu.blcu.edu.cn (J.Z.); 202421198242@stu.blcu.edu.cn (F.H.); 2 Institute of Life and Health Sciences, Beijing Language and Culture University, Beijing 100083, China; 3 Key Laboratory of Language and Cognitive Science (Ministry of Education), Beijing Language and Culture University, Beijing 100083, China; 4 School of Chinese Language and Literature, Beijing Normal University, Beijing 100875, China

**Keywords:** emotional prosody, Mandarin Chinese, background masking, speech-shaped noise, multi-talker babble, confusion structure, Net Flow

## Abstract

Emotional prosody is a key cue for affective communication, yet everyday speech often occurs in background noise. It remains unclear whether masking only reduces Mandarin emotional prosody recognition accuracy or also alters emotion-confusion structure. We examined this issue with speech-shaped noise (SSN) and multi-talker babble (MTB). Thirty-six native Mandarin-speaking young adults identified semantically neutral sentences conveying happiness, sadness, anger, and surprise in silence and under SSN and MTB at 0 dB and −4 dB signal-to-noise ratios. Recognition accuracy was analyzed using trial-level generalized linear mixed-effects models, whereas confusion structure was assessed using confusion matrices, sadness–high-arousal boundary errors, and Net Flow, an index of directed target–response asymmetry. Accuracy declined under masking relative to silence, with the poorest performance in the MTB −4 dB condition. Across emotions, estimated accuracy was lowest for surprise and comparatively high for sadness. The MTB −4 dB condition increased sadness–high-arousal boundary errors; Net Flow characterized a source-like pattern for surprise and a more sink-like pattern for sadness, particularly in this condition. These findings suggest that background masking affects Mandarin emotional prosody perception not only by reducing recognition accuracy but also by altering directed response-error structure.

## 1. Introduction

Emotional prosody is a central source of affective information in spoken communication. Beyond lexical meaning, listeners infer a speaker’s emotional state and communicative intent through the integration of multiple acoustic dimensions, including fundamental frequency (F0), intensity, temporal structure, and voice quality (Banse & Scherer, 1996; Juslin & Laukka, 2003; Scherer, 2003). Because these cues are probabilistic rather than category-specific, vocal emotion decoding depends not only on the cue combinations themselves but also on the listening conditions in which they are heard. Although listeners can recognize many vocally expressed emotions above chance across languages and tasks (Juslin & Laukka, 2003; Pell et al., 2009), everyday affective communication often takes place in background noise. Under masking, the availability and reliability of emotion-relevant cue combinations may decrease, raising the question of whether background masking merely lowers recognition accuracy or also alters the structure of emotion confusions.

Masking effects may differ across emotion categories because emotional categories are separated by overlapping but partially diagnostic acoustic configurations. High-arousal emotions such as anger and happiness are generally associated with increased vocal activation, whereas sadness is characterized by relatively lower-arousal vocal patterns (Banse & Scherer, 1996; Juslin & Laukka, 2003; Scherer, 1986). However, acoustic cues do not map onto discrete emotion categories in a one-to-one way; emotional prosody is based on combinations of cues rather than isolated acoustic parameters (Bänziger & Scherer, 2005; Larrouy-Maestri et al., 2025; Scherer & Bänziger, 2004). Thus, by reducing the perceptual distinctiveness of emotion-related acoustic configurations, background masking may produce category-specific vulnerability and structured confusion patterns rather than a uniform decline across all emotions.

The extent and form of masking-related disruption should depend on masker type and signal-to-noise ratio (SNR) (Mattys et al., 2012). For instance, speech-shaped noise (SSN) typically matches the long-term average spectrum of speech and is used as a relatively steady spectral masker, whereas multi-talker babble (MTB) contains temporal fluctuations, periodicity, and competing talker structure. MTB therefore provides a more complex background masker that can involve both energetic overlap and informational masking. Informational masking can arise from target–masker similarity, stream selection, and attention drawn to competing speech (Brungart, 2001; Durlach et al., 2003; Kidd et al., 2008; Rosen et al., 2013). These speech-like masker properties may be especially relevant for emotional prosody because listeners must integrate dynamic patterns of acoustic information from the target speaker over the utterance. SNR can further shape these effects by changing the relative availability of target emotion cues, with lower SNR reducing access to the acoustic information needed for category separation. Comparing SSN and MTB across SNR levels can therefore test whether speech-like competition at lower SNR produces stronger disruption of emotion-relevant cue combinations than a relatively steady speech-shaped masker.

These issues are especially relevant in Mandarin Chinese, where F0 variation contributes to both lexical tone contrasts and emotional prosody. This makes Mandarin a theoretically informative case for examining emotional prosody under masking, because affective cues may rely partly on acoustic dimensions that also carry linguistic contrast. Under masking, Mandarin lexical tone perception can remain relatively robust in SSN and MTB, although performance varies with SNR and, within babble, the number of competing talkers (Wang & Xu, 2020). Emotional prosody, however, may be less robust than lexical tone in babble noise. For example, Zhang et al. (2023) found that Mandarin listeners identified lexical tones more accurately and quickly than vocal emotions in MTB. Together, these findings suggest that Mandarin lexical tone and emotional prosody are acoustically linked but may not be equally robust under adverse listening conditions.

Prior Mandarin work has shown that emotional prosody recognition is sensitive to listening context and emotion category, but systematic evidence across masker type and SNR remains limited. For example, Kuang et al. (2024) showed that Mandarin emotional prosody recognition in children and adults was influenced by babble noise, prosody–semantics congruity, working memory, and age. Liu and Pell (2012) further showed that Mandarin vocal emotions differ in recognizability, with positive or high-arousal categories, including happiness and pleasant surprise, identified less consistently than categories such as sadness or anger. This pattern motivated the sadness–surprise contrast used to examine directional asymmetry in the present design. What remains unclear is whether systematic variation in masker type and SNR affects Mandarin emotional prosody uniformly across emotion categories or instead produces category-specific vulnerability and structured confusions in semantically controlled materials.

Accuracy alone cannot determine whether masking-induced errors reflect random performance degradation or systematic changes in emotion-category relationships. The same decrease in accuracy may arise from diffuse guessing, increased confusion between specific emotion categories, or asymmetric response tendencies in which some categories attract misidentifications from others. Such confusion patterns provide information about perceived relationships among emotion categories and whether masking alters category boundaries. This approach is consistent with accounts of emotion perception that incorporate both discrete categories and graded affective structure. Russell’s (1980) circumplex model organizes affective concepts along valence and arousal dimensions, while more recent work suggests that vocal emotion recognition involves both discrete categories and graded relations among them (Cowen et al., 2019). Within the emotion set examined here, sadness served as the relatively low-arousal category, whereas happiness, anger, and surprise were treated as relatively higher-arousal categories. Because emotional prosody conveys arousal-related information through dynamic vocal cues, speech-like masking at lower SNR may reduce the perceptual distinction across this low- versus higher-arousal boundary. Therefore, MTB at −4 dB was expected to increase sadness–high-arousal boundary-crossing confusions rather than simply reduce recognition accuracy uniformly.

Beyond identifying which categories are confused, the direction of target–response confusions can characterize whether particular emotions tend to be misidentified as others or instead attract misidentifications from other targets. In this sense, an emotion may show a source-like pattern when it is often confused with other responses, or a sink-like pattern when it attracts errors from other targets. Given prior Mandarin evidence that the related category of pleasant surprise is less consistently recognized than sadness or anger (Liu & Pell, 2012), the sadness–surprise contrast was selected as an a priori comparison for examining directional asymmetry. This focal comparison was embedded within the broader four-emotion confusion network and was not intended to represent all directed relations among emotions. Standard confusion matrices show target-to-response probabilities, whereas directed network measures can summarize these directional asymmetries more compactly across the four emotion nodes. Network approaches have been used to represent relations among categories and concepts in cognitive research and can be adapted to characterize directed relations among emotion-confusion responses (Baronchelli et al., 2013; Siew et al., 2019). This approach allows masking-related changes in both the amount and direction of confusion to be considered within the same target–response structure.

Accordingly, the present study examined how Mandarin emotional prosody recognition and confusion structure vary across listening conditions. Young adult native speakers of Mandarin identified semantically neutral Mandarin sentences spoken to express happiness, sadness, anger, and surprise in silence and in two masker types, SSN and MTB, at two SNR levels. We assessed recognition accuracy and target–response confusions as two complementary aspects of emotional prosody perception. Confusion structure was further examined through boundary-error analyses and directional analyses of target–response choices, with the sadness–surprise contrast serving as the focal Net Flow comparison. This approach allowed us to test whether background masking reduces recognition accuracy alone or also alters the structure and directionality of emotion confusions.

The specific research questions (RQs) and corresponding hypotheses (Hs) were organized around two analytic aims: recognition accuracy and confusion structure.

**RQ1:**  *How does listening condition affect emotional prosody recognition accuracy in young Mandarin-speaking adults, and do these effects vary across emotion categories?*

**H1:**  *Recognition accuracy was expected to decline under masking relative to quiet listening and to be poorer under MTB than under SSN, especially at the lower SNR level. These effects were also expected to show variation across emotion categories, reflecting possible differences in recognition performance under degraded listening conditions.*

**RQ2:**  *Does background masking alter the structure of emotional prosody confusions beyond its effects on recognition accuracy, as reflected in boundary-crossing errors and directional target–response asymmetry?*

**H2:**  *Because MTB combines speech-like competition with reduced target-cue availability at the lower SNR level, MTB at −4 dB was expected to produce the strongest increase in sadness–high-arousal boundary errors relative to the other listening conditions. It was also expected to alter directional target–response asymmetry, especially in the sadness–surprise focal contrast.*

## 2. Materials and Methods

### 2.1. Participants

Thirty-six young adults were recruited from the university student community using convenience sampling. The sample included 24 females and 12 males. Participants were between 18 and 27 years of age (*M* = 21.97, *SD* = 2.72). All participants self-identified as native speakers of Mandarin Chinese and reported using Mandarin as their primary language in daily communication. Most participants reported Putonghua as their primary Mandarin variety (*n* = 29); the remaining participants reported Southwestern Mandarin varieties (*n* = 4), an unspecified regional Mandarin variety (*n* = 2), or Shandong Mandarin (*n* = 1). Systematic information regarding participants’ non-Chinese language experience was not collected. All participants self-reported normal or corrected-to-normal vision, no known hearing impairment, and no history of neurological or psychiatric disorders; no audiometric screening was conducted. All participants provided written informed consent before the experiment and received compensation after participation. No participants were excluded, and the final analyzed sample therefore included all 36 recruited participants. Participant-level exclusion criteria and trial-level preprocessing procedures are described in Section 2.4.1.

A design-level sample-size adequacy check was conducted in G*Power 3.1.9.7 (Faul et al., 2009) using a repeated-measures ANOVA approximation. This approach was selected because the primary experimental design involved within-subject manipulations of listening condition and target emotion. The design was therefore specified as one group with 20 repeated measurements, corresponding to the 5 listening conditions crossed with the 4 target emotions. The calculation used a small-to-moderate Cohen’s *f* of 0.15, an α level of 0.05, power of 0.90, a repeated-measures correlation of 0.50, and an epsilon correction of 0.75. The required sample size was 36 participants, with an actual power of 0.909. This calculation provided a priori design-level justification for the final sample size. Because the subsequent analyses used trial-level models that incorporate participant- and item-level variability, this calculation should not be interpreted as an exact power analysis for the final statistical models.

### 2.2. Stimuli

#### 2.2.1. Speech Stimulus Selection and Validation

The speech stimuli were selected from the CASIA Chinese Natural Emotional Audio–Visual Database (CHEAVD; Li et al., 2017), a natural Chinese emotion database developed for affective speech and multimodal emotion research. The recordings had a sampling rate of 16 kHz and 16-bit resolution. The corpus consists of acted emotional speech produced by professional speakers.

To reduce potential semantic bias in the emotion identification task, sentence texts were screened before the final speech tokens were selected. Five native Mandarin-speaking raters with relevant training in language or speech research evaluated 50 candidate sentence texts from the corpus. Each text was rated on a 7-point semantic valence scale, with 1 indicating very negative, 4 neutral, and 7 very positive. Inter-rater reliability was assessed using a two-way mixed-effects, average-measures intraclass correlation coefficient. Agreement among raters was high: ICC(3,k) = 0.82, *F*(49,196) = 5.50, *p* < 0.001. Based on these ratings, 24 sentence texts with neutral semantic content and high rater agreement were selected. The selected texts followed a subject–verb–object structure and were comparable in length, with each sentence containing five to six Chinese characters.

Candidate speech tokens selected for validation were drawn from four speakers, including two male and two female speakers, and represented four emotion categories: happiness, sadness, anger, and surprise. A total of 340 candidate recordings were submitted to perceptual validation. The validation experiment was conducted with 16 native Mandarin-speaking listeners, including 9 females, none of whom participated in the formal experiment. Listeners completed a four-alternative forced-choice emotion identification task. On each trial, they identified the perceived emotion of a speech token by choosing one of the four emotion labels. Only recordings with item-level recognition accuracy of at least 0.75 were considered eligible for inclusion in the final stimulus set.

From the validated recordings, 64 target speech tokens were selected for the formal experiment using a speaker-balanced structure. The final set was balanced across both emotion categories and speakers: each emotion category included 16 tokens, with four tokens produced by each of the four speakers, reducing the possibility that any emotion category was represented disproportionately by a particular speaker. An additional 12 validated recordings were selected as practice stimuli. Descriptive acoustic characteristics of the final clean target tokens, including duration and F0-related measures, are summarized by emotion category in Table S1.

#### 2.2.2. Noise Generation and Stimulus Construction

All speech tokens were processed in MATLAB R2024b (MathWorks, Natick, MA, USA) before stimulus construction. Leading and trailing silent portions were removed using an energy-based endpoint detection procedure. A frame was classified as part of the effective speech portion only when it met both threshold criteria for at least five consecutive frames: a level above −30 dB relative to the peak level and an energy value above the 20th percentile of the utterance-level energy distribution. To preserve low-energy phonetic information, including voiceless consonants and final decay portions, safety margins were applied by retaining 10 ms before the detected onset and 100 ms after the detected offset. The resulting signals were converted to mono, resampled to 44.1 kHz, and peak-normalized to prevent clipping during subsequent processing.

The effective speech portions were then root-mean-square (RMS) normalized to reduce level differences across tokens. During normalization, the maximum absolute amplitude was limited to 0.95 to avoid clipping. After level normalization, 300 ms of digital silence was added before and after the effective speech portion. This ensured a consistent temporal baseline and allowed the masker to begin before speech onset and continue after speech offset in the noise conditions (Rosen et al., 2013; Wang & Xu, 2020; Zhang et al., 2023).

Two types of background maskers were used: SSN and MTB. The SSN was generated to match the long-term average speech spectrum of the target speech tokens (Rosen et al., 2013; Wang & Xu, 2020). The normalized speech tokens were concatenated into a long-duration speech sample. The long-term average spectrum was estimated using Welch’s power spectral density method with a 2048-sample Hamming window, 50% overlap, and a 4096-point fast Fourier transform. The resulting spectrum was smoothed with an approximately 100 Hz moving-average filter and used to shape Gaussian white noise. This produced a 60 s monaural SSN template at a sampling rate of 44.1 kHz. As shown in Figure S1, the generated SSN closely matched the long-term average spectrum of the target speech tokens, with Pearson’s *r* > 0.99 between the smoothed normalized spectra over 50–8000 Hz. The SSN template was RMS-normalized before SNR mixing.

The MTB was constructed as a six-talker Mandarin babble masker from emotionally neutral speech samples in CSEMOTIONS, a separate Mandarin speech dataset introduced as part of the Marco-Voice framework (Tian et al., 2026). Speech samples from six speakers, including three male and three female speakers who did not appear in the target stimulus set, were used to create a 60 s continuous babble template. The MTB materials did not contain target-corpus recordings, target speakers, or target sentences. This separation was intended to reduce direct target overlap and potential confounding arising from shared speakers or recordings. The MTB was included as a speech-like background masker that can involve both energetic and informational masking (Rosen et al., 2013; Wang & Xu, 2020; Zhang et al., 2023). To ensure stable acoustic energy and a constant number of active talkers from the beginning of the masker segment, the talker streams were initialized before the nominal onset of the babble. This avoided the gradual energy build-up that can occur when talker streams are introduced only after stimulus onset. The resulting MTB template was RMS-normalized before SNR mixing.

For the formal experiment, each of the 64 target speech tokens was presented in five listening conditions: silence, SSN at 0 dB SNR, SSN at −4 dB SNR, MTB at 0 dB SNR, and MTB at −4 dB SNR. In each noise condition, a segment of the corresponding 60 s noise template was randomly selected for each trial. Noise segments were sampled independently across trials, including trials containing the same target speech token in different noise conditions. The masker began 300 ms before the onset of the effective speech portion and ended 300 ms after its offset. The RMS level of the speech token was kept constant, and the RMS level of the masker segment was adjusted to achieve the designated SNR. SNR was calculated in dB from the RMS levels of the effective speech portion and the corresponding masker segment over the effective speech interval. After target–masker mixing, the peak amplitude of the mixed waveform was checked, and proportional scaling was applied when necessary to prevent clipping while preserving the intended SNR. A 50 ms raised-cosine ramp was applied to the onset and offset of the final waveform. The final set contained 320 experimental stimuli, corresponding to 64 target speech tokens presented across five listening conditions.

### 2.3. Procedure

The experiment used a 4 × 5 within-subject design, crossing target emotion (happiness, sadness, anger, and surprise) with listening condition (silence, SSN at 0 dB SNR, SSN at −4 dB SNR, MTB at 0 dB SNR, and MTB at −4 dB SNR). Testing was conducted in a quiet laboratory room, and auditory stimuli were presented binaurally through EDIFIER K820NC headphones (Edifier International Limited, Dongguan, China). During practice, each participant adjusted the playback volume to a comfortable and clearly audible level, which was then held constant throughout the formal experiment. Because target–masker ratios had been set digitally during stimulus construction, this adjustment changed only the overall playback level and did not alter the intended SNR within each noise condition. Participants completed a four-alternative forced-choice emotion identification task. Stimulus presentation and response collection were controlled using PsychoPy version 2021.1.4 (Peirce et al., 2019).

Before the formal experiment, participants completed three short practice blocks in the order of silence, SSN, and MTB to familiarize themselves with the task and the listening conditions. Each practice block contained four trials using stimuli not included in the formal task. The formal experiment comprised 320 trials divided into five 64-trial blocks, with each block corresponding to one listening condition. This blocked design was adopted to maintain a stable listening context and minimize frequent switching between masker conditions. The order of the five listening-condition blocks was counterbalanced across participants using a Latin-square scheme, and block position was included as a covariate in the accuracy model. A 1 min break was provided between blocks.

Each trial began with a central fixation cross for 500 ms, after which the auditory stimulus was presented together with four on-screen response options. The options displayed emotion labels, facial-expression icons, and key prompts for the A, S, K, and L response keys. Participants were instructed to identify the perceived emotion as accurately and quickly as possible, and responses could be made during or after stimulus presentation. The mapping between emotion categories and response keys was counterbalanced across participants using a Latin-square scheme and remained fixed for each participant. Within each block, trials were pseudorandomized to reduce immediate repetitions of the same target emotion. The inter-trial interval varied between 500 and 1000 ms randomly. Accuracy and reaction times (RTs), measured from the onset of the auditory stimulus, were recorded for each trial. RTs were used only for preprocessing and were not analyzed as outcome measures in the present study.

### 2.4. Data Analysis

#### 2.4.1. Data Preprocessing

The raw dataset included 11,520 trials from 36 participants, with 320 trials per participant. Trials without a response were removed. We further excluded trials with RTs shorter than 200 ms or longer than 10,000 ms. The 200 ms lower cutoff was used as a practical safeguard to exclude extremely rapid responses that may reflect anticipatory or accidental key presses. Because the task required whole-sentence prosody identification, this threshold should be interpreted as a permissive screening criterion rather than an estimate of the minimum time required for valid emotion recognition. A within-participant RT outlier screen then removed trials falling outside ±3 SD of each participant’s log-transformed RT distribution. These preprocessing steps excluded 96 trials in total, corresponding to 0.83% of the raw data, leaving 11,424 valid trials. Participant-level response patterns were then screened for evidence of random responding, strong response bias, or disengagement. Because the task used a four-alternative forced-choice format, chance performance was 25%. Participants were flagged for exclusion if they met any of three participant-level criteria: overall accuracy at or below chance, more than 70% of valid responses assigned to a single response category, or fewer than 90% of trials remaining after preprocessing. No participant met these criteria, and all 36 participants were retained.

Trial accuracy was coded as a binary variable indicating whether the reported emotion matched the target emotion. Target and response labels were retained as four-category emotion variables for subsequent analyses of accuracy and confusion patterns.

#### 2.4.2. Statistical Analysis

Statistical analyses were organized around the two research questions. RQ1 was addressed using trial-level models of recognition accuracy, with listening condition and target emotion as the primary predictors. RQ2 was addressed by characterizing target–response confusion structure through two complementary analyses: boundary-error analysis, which examined where errors were concentrated, and Net Flow analysis, which examined directional asymmetry in target–response choices.

Recognition Accuracy Analysis. For RQ1, recognition accuracy was analyzed using generalized linear mixed-effects models (GLMM) with a binomial error distribution and a logit link function. Accuracy was coded as 1 for correct responses and 0 for incorrect responses. This model accommodated the binary response outcome while estimating participant- and item-level variability (Baayen et al., 2008).

The model included listening condition, target emotion, and their interaction as fixed effects. Standardized block position was included as a covariate to account for changes in performance over the course of the experiment. Listening condition was treated as a five-level factor, consisting of a silence baseline and four noise conditions. The four noise conditions formed a 2 × 2 design crossing masker type, SSN versus MTB, with SNR, 0 dB versus −4 dB. Planned orthogonal contrasts were used to test a priori comparisons motivated by the design (Schad et al., 2020). These contrasts captured the overall effect of noise relative to silence, the effect of masker type, the effect of SNR, and the masker type × SNR interaction, as well as whether these effects differed across target emotions. Listening condition was therefore decomposed into four contrasts: noise versus silence, MTB versus SSN, 0 dB versus −4 dB SNR, and the masker type × SNR interaction. Condition levels were ordered as silence, SSN 0 dB, SSN −4 dB, MTB 0 dB, and MTB −4 dB. The planned contrasts were coded as follows: noise versus silence [−4, 1, 1, 1, 1], MTB versus SSN [0, −1, −1, 1, 1], 0 dB versus −4 dB SNR [0, 1, −1, 1, −1], and the masker type × SNR interaction [0, −1, 1, 1, −1]. Target emotion was coded using three orthogonal contrasts: happiness versus sadness [1, −1, 0, 0], happiness/sadness versus anger [1, 1, −2, 0], and happiness/sadness/anger versus surprise [1, 1, 1, −3], with emotion levels ordered as happiness, sadness, anger, and surprise.

Random-effects structures were evaluated following recommendations for mixed-effects modeling (Barr et al., 2013; Matuschek et al., 2017). Candidate models included random intercepts for participants and items, by-participant random slopes for listening condition and target emotion, and by-item random slopes for listening condition. The original recording was used as the item-level grouping factor because each recording was presented across the five listening conditions. By-item slopes for target emotion were not fitted because each item belonged to only one target-emotion category. Random slopes were fitted without correlation parameters to improve model stability. The selected model converged without a singular fit. It included by-participant random slopes for listening condition and target emotion, and by-item random slopes for listening condition. Model 1 was specified as follows:

Accuracy ~ Listening Condition × Emotion + Block + (1 + Listening Condition + Emotion || Participant) + (1 + Listening Condition || Item).

In this formula, Listening Condition refers to the five-level listening-condition factor described above, and the random slopes for Listening Condition and Emotion were implemented using the planned orthogonal contrasts. The Listening Condition × Emotion interaction tested whether the effects of noise, masker type, SNR, and their interaction differed across target emotions. Fixed effects were tested using Type III Wald χ^2^ tests. Estimated marginal means (EMMs) on the response scale and Bonferroni-corrected pairwise comparisons were used to characterize fixed-effect patterns and condition-specific differences. Odds ratios and 95% confidence intervals were calculated by exponentiating the fixed-effect estimates and their confidence limits.

Confusion Structure and Network Flow. Whereas the accuracy analysis tested whether masking affected recognition performance, RQ2 examined whether masking altered the structure of emotional prosody confusions. Target-to-response confusions were first summarized using confusion matrices. For each listening condition, responses were summarized as row-normalized conditional probabilities, representing the probability of each response emotion given a target emotion. These matrices provided the basis for the boundary-error and Net Flow analyses described below.

To test whether masking changed the composition of emotion confusions, incorrect responses were classified as sadness–high-arousal boundary errors or within-high-arousal errors. For this design-based contrast, happiness, anger, and surprise were grouped as relatively higher-arousal emotions, whereas sadness was treated as the relatively lower-arousal emotion. Sadness–high-arousal boundary errors included both directions of confusion under this grouping: sadness responses to relatively higher-arousal targets and higher-arousal responses to sadness targets. Within-high-arousal errors were defined as incorrect responses among happiness, anger, and surprise. The boundary-error measure was defined as the proportion of theoretically relevant errors that crossed the sadness–high-arousal boundary:

P_boundary_ = *B*/(*B* + *W*)

where *B* denotes the number of sadness–high-arousal boundary errors and *W* denotes the number of within-high-arousal errors for a given participant and listening condition. Higher values indicate that errors were more strongly concentrated along the sadness–high-arousal boundary.

The boundary-error measure was analyzed using Model 2, a binomial GLMM with a logit link. For each participant and listening condition, the model estimated the probability that an included error was a sadness–high-arousal boundary error, with B modeled as the number of successes and *W* as the number of failures. Listening condition was entered as a fixed effect, with random intercepts for participants. Model 2 was specified as follows:

P_boundary_ ~ Listening Condition + (1 | Participant).

Listening condition was coded using the same planned orthogonal contrasts as in the recognition-accuracy analysis: noise versus silence, MTB versus SSN, 0 dB versus −4 dB SNR, and the masker type × SNR interaction. Because unmodeled heterogeneity can produce overdispersion in binomial mixed-effects models, overdispersion was evaluated as the Pearson χ^2^ statistic divided by the residual degrees of freedom; values close to 1 indicated no substantial overdispersion (Bolker et al., 2009; Harrison, 2015). The binomial GLMM was retained because the overdispersion ratio was below the commonly used threshold of 1.5. Fixed effects were tested using Type III Wald χ^2^ tests. EMMs on the response scale and Bonferroni-corrected pairwise comparisons were used to characterize differences among listening conditions in the probability of sadness–high-arousal boundary errors.

Beyond confusion composition, we examined whether masking changed confusion directionality. For this purpose, the row-normalized confusion matrices were represented as directed weighted networks (Siew et al., 2019). The four emotions served as nodes, and off-diagonal conditional confusion probabilities served as directed edges from target emotions to response emotions. Group-level networks were visualized separately by listening condition, with edge width scaled by confusion probability. For visualization, only edges with probabilities of at least 0.05 were displayed.

Directional organization was quantified using Net Flow. For a given emotion node, out-strength was defined as the summed probability that the target emotion was misidentified as other response emotions, whereas in-strength was defined as the summed probability that other target emotions were misidentified as that response emotion. Net Flow for each emotion node was computed as

Net Flow = In-strength − Out-strength.

Positive Net Flow was interpreted as a sink-like node pattern, indicating more incoming than outgoing misidentifications; negative Net Flow indicated a source-like pattern, reflecting more outgoing than incoming misidentifications. The full four-node networks were used for descriptive visualization. For the primary inferential Net Flow model, sadness and surprise were selected as focal nodes. This contrast examined directional asymmetry between the relatively lower-arousal category and surprise, a higher-arousal category expected to show greater vulnerability based on prior Mandarin evidence (Liu & Pell, 2012).

Participant-level Net Flow values for sadness and surprise were analyzed using Model 3, a linear mixed-effects model (LMM). Model 3 included listening condition, focal emotion node, and their interaction as fixed effects, with random intercepts for participants. This random-intercept structure was used because Net Flow was computed as a participant-level network summary within each listening condition, and the model was intended to test condition- and node-level differences in these summary indices. Model 3 was specified as follows:

Net Flow ~ Listening Condition × Focal Emotion Node + (1 | Participant).

The same planned contrasts for listening conditions were used. Fixed effects were evaluated using Type III Wald χ^2^ tests. For Model 3, EMMs and Bonferroni-corrected pairwise comparisons were used to characterize listening-condition effects within each focal emotion node and focal-node differences within each listening condition. A complementary four-node Net Flow sensitivity analysis was conducted using Model 4. This model used the same fixed-effect structure as Model 3 but included Net Flow values for all four emotion nodes. It assessed whether the focal sadness–surprise pattern was consistent with the broader four-emotion network structure. Because Net Flow values across all nodes within each participant-by-condition network sum to zero, Model 4 was interpreted as a robustness check of the focal-node analysis rather than as independent node-level evidence.

All analyses and visualizations were conducted in R version 4.3.2 (R Foundation for Statistical Computing, Vienna, Austria; R Core Team, 2023). Mixed-effects models were fitted using lme4 and lmerTest (Bates et al., 2015; Kuznetsova et al., 2017), with the bobyqa optimizer used where necessary to facilitate convergence. Type III Wald χ^2^ tests were performed using car (Fox & Weisberg, 2019). EMMs and pairwise comparisons were obtained using emmeans (Lenth, 2023), with Bonferroni correction applied where appropriate. Directed confusion networks were constructed and visualized using igraph and ggraph (Csardi & Nepusz, 2006; Pedersen, 2022), and figures were created using ggplot2 (Wickham, 2016).

## 3. Results

Results are presented in two parts corresponding to the two RQs. We first report recognition accuracy across listening conditions and target emotions, addressing RQ1. We then report analyses of confusion structure, addressing RQ2. Within this second aim, boundary-error analyses examine the distribution of errors across emotion categories, whereas Net Flow analyses examine directional asymmetry in target–response choices.

### 3.1. Recognition Accuracy Across Listening Conditions and Emotions

Recognition accuracy was analyzed using Model 1, a trial-level binomial GLMM. Type III Wald χ^2^ tests for Model 1 showed significant effects of listening condition (χ^2^(4) = 71.250, *p* < 0.001) and target emotion (χ^2^(3) = 49.403, *p* < 0.001; Table 1). The listening condition × target emotion interaction did not reach conventional statistical significance (χ^2^(12) = 21.008, *p* = 0.050). Block position was also significant (χ^2^(1) = 40.533, *p* < 0.001), suggesting that recognition accuracy changed over the course of the experiment.

Planned orthogonal contrasts showed that accuracy was lower in noise than in silence, lower in MTB than in SSN, and lower at −4 dB than at 0 dB SNR (Table 1). The masker type × SNR contrast was also significant, indicating a larger decrease from 0 dB to −4 dB SNR in MTB than in SSN. Model-estimated marginal means showed that recognition accuracy was highest in silence and lowest in MTB at −4 dB (Table S2). Averaged across target emotions, estimated accuracy was 0.905 in silence, 0.876 in SSN at 0 dB, 0.849 in SSN at −4 dB, 0.864 in MTB at 0 dB, and 0.766 in MTB at −4 dB.

**Figure 1:**
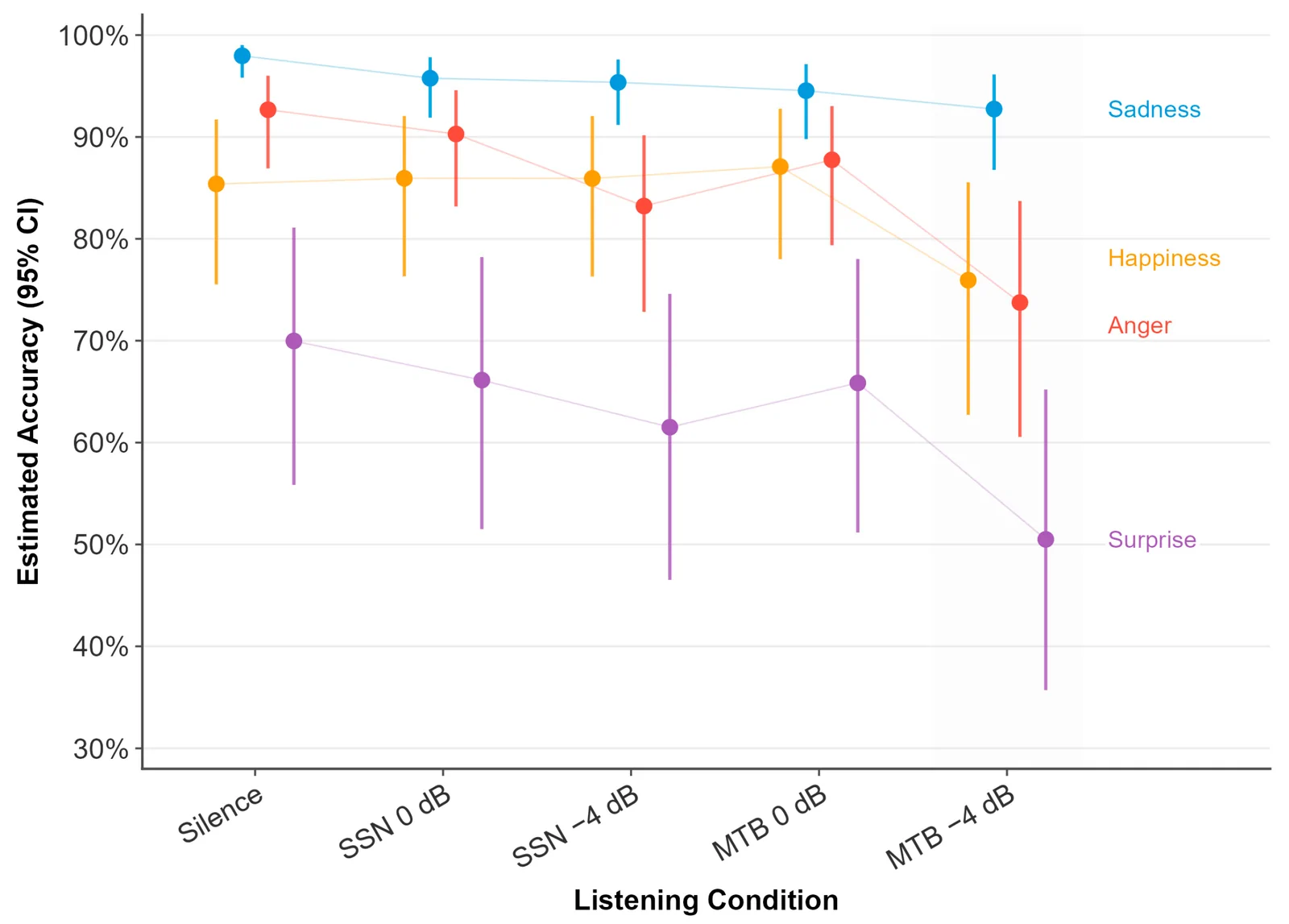
Estimated recognition accuracy by listening condition and target emotion. Points represent estimated marginal means from the trial-level binomial GLMM. Error bars represent 95% confidence intervals on the response-probability scale. SSN = speech-shaped noise; MTB = multi-talker babble. GLMM = generalized linear mixed-effects model.

Given the marginal interaction, condition-specific emotion patterns are presented as descriptive patterns rather than interpreted as strong evidence of differential masking effects across emotions (Figure 1). Averaged across listening conditions, sadness had the highest estimated accuracy and surprise had the lowest, with happiness and anger falling between these two categories (Table S2). This pattern was most apparent in MTB −4 dB, where estimated accuracy was lowest for surprise (0.505) but remained comparatively high for sadness (0.927).

### 3.2. Confusion Structure Under Masking

#### 3.2.1. Target–Response Confusion Patterns

**Figure 2:**
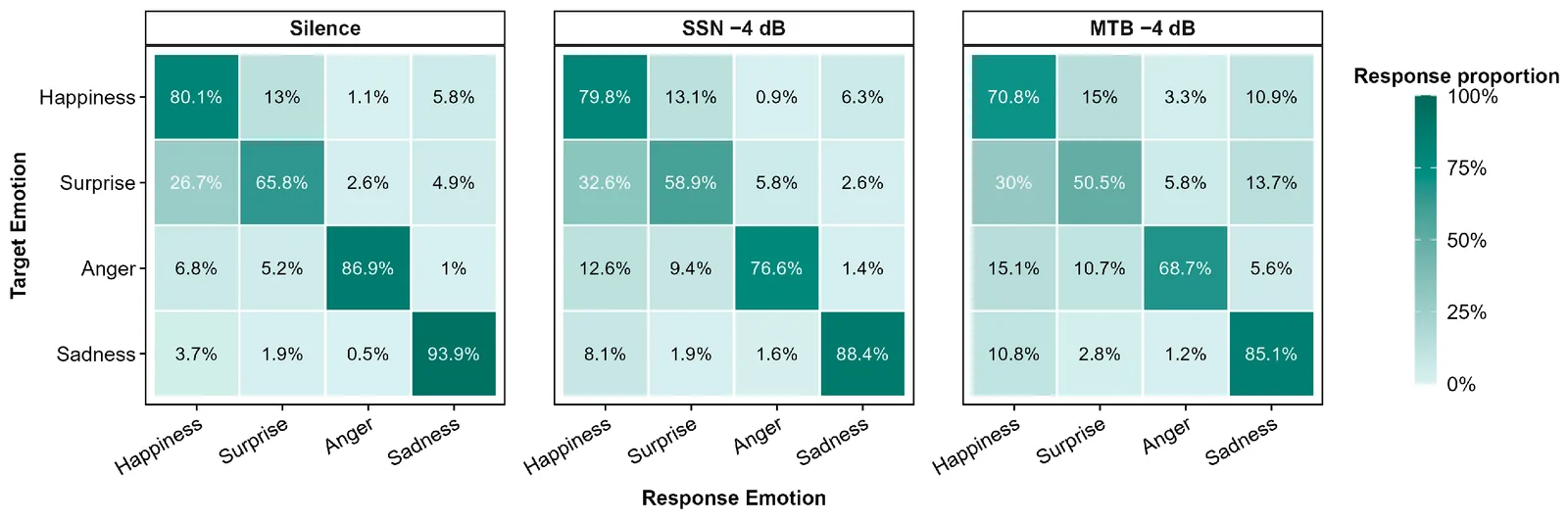
Target–response confusion matrices for silence, SSN −4 dB, and MTB −4 dB. Rows represent target emotions, and columns represent response emotions. Cell values are row-normalized response proportions, reflecting the probability of each response emotion given each target emotion. Darker shading indicates higher response proportions. Complete matrices for all listening conditions are provided in Table S3. SSN = speech-shaped noise; MTB = multi-talker babble.

Target–response confusion matrices were used to summarize response patterns across target and response emotions. The row-normalized matrices showed that errors were not evenly distributed across response emotions (Figure 2; Table S3). Among the conditions shown in Figure 2, surprise targets were frequently identified as happiness, with surprise-to-happiness response proportions of 0.267 in silence, 0.326 in SSN −4 dB, and 0.300 in MTB −4 dB.

**Figure 3:**
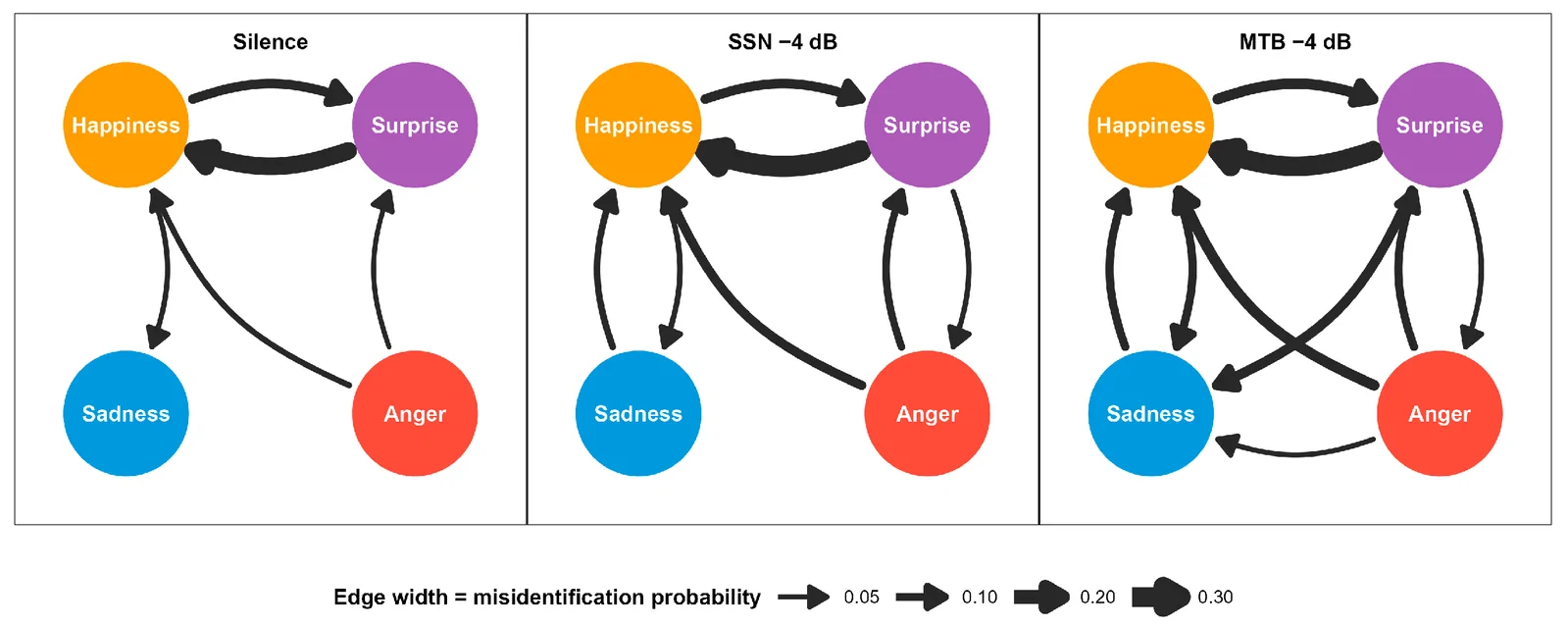
Directed target-to-response confusion networks for silence, SSN −4 dB, and MTB −4 dB. Nodes represent emotion categories, and directed edges represent off-diagonal confusions from target emotion to response emotion. Edge width is proportional to the row-normalized conditional probability of each confusion. Only edges with probabilities of at least 0.05 are displayed. SSN = speech-shaped noise; MTB = multi-talker babble.

To further examine the directionality of these confusions, row-normalized off-diagonal probabilities were visualized as directed confusion networks, with edges representing target-to-response confusions (Figure 3). The networks highlighted two patterns relevant to RQ2: surprise showed strong outgoing edges, particularly toward happiness, and MTB −4 dB showed stronger incoming edges to sadness than silence. For example, surprise-to-sadness confusions increased from 0.049 in silence to 0.137 in MTB −4 dB. These patterns were examined further in the boundary-error and Net Flow analyses below.

#### 3.2.2. Sadness–High-Arousal Boundary Errors

**Figure 4:**
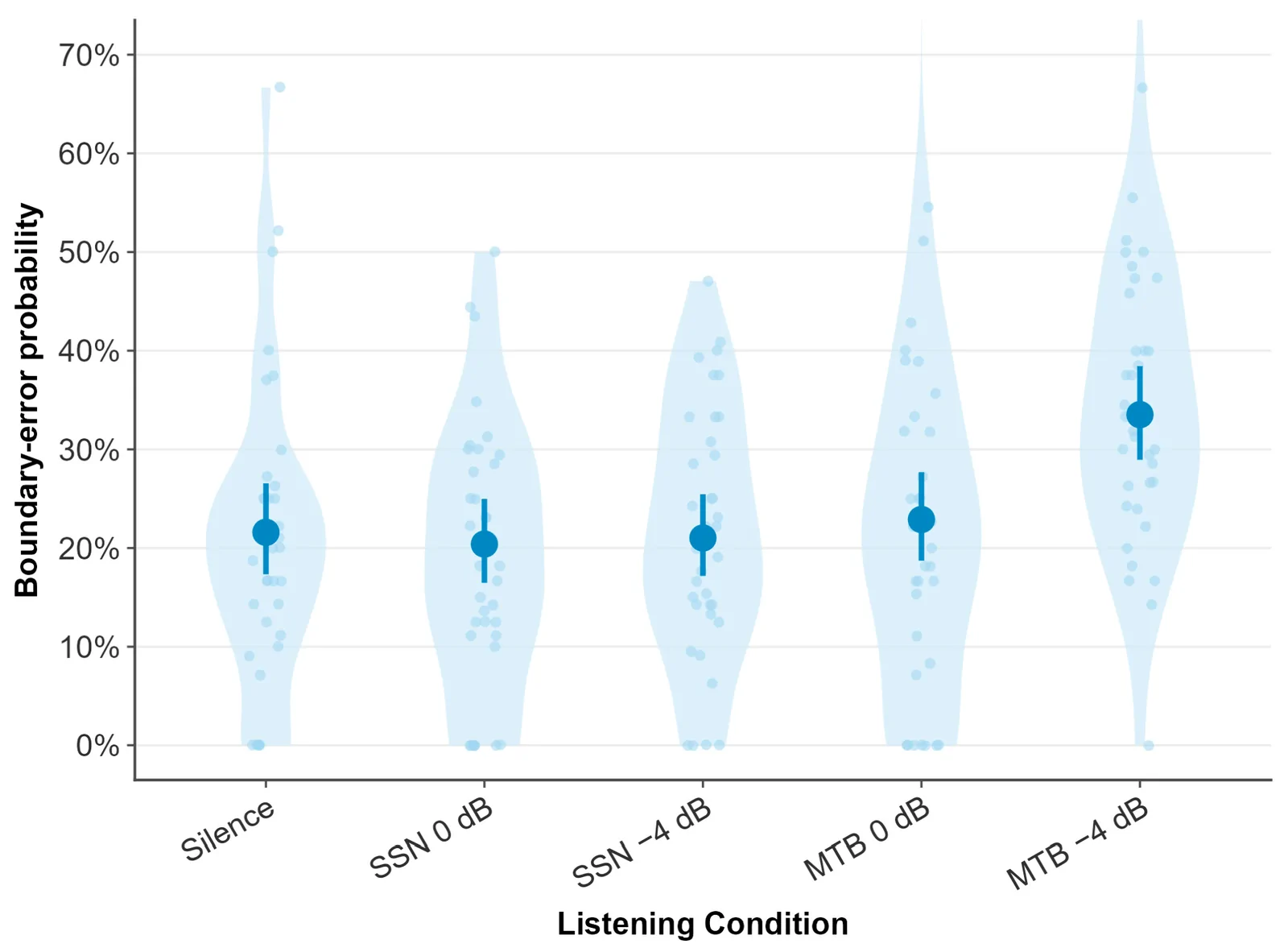
Estimated probability of sadness–high-arousal boundary errors by listening condition. Shaded distributions and light points show participant-level observed proportions. Large points represent estimated marginal means from the boundary-error mixed-effects model, and error bars represent 95% confidence intervals on the response-probability scale. Sadness–high-arousal boundary errors were defined as bidirectional confusions between sadness and the relatively higher-arousal emotions, including happiness, anger, and surprise. SSN = speech-shaped noise; MTB = multi-talker babble.

The boundary-error analysis tested whether listening condition changed the probability of a sadness–high-arousal boundary error. Model 2 showed no evidence of problematic overdispersion (Pearson overdispersion ratio = 0.816), supporting the use of the binomial GLMM. Type III Wald χ^2^ tests showed a significant effect of listening condition on sadness–high-arousal boundary errors (χ^2^(4) = 40.602, *p* < 0.001; Table 2), with the proportion of boundary-crossing errors varying across listening conditions. Planned contrasts showed no significant overall difference between noise and silence (*p* = 0.257), but significant effects of masker type (*p* < 0.001), SNR (*p* = 0.005), and the masker type × SNR interaction (*p* = 0.014). Model-estimated marginal means from Model 2 showed the highest boundary-error probability in MTB −4 dB (Figure 4), with estimated probabilities of 0.216 in silence, 0.204 in SSN 0 dB, 0.210 in SSN −4 dB, 0.229 in MTB 0 dB, and 0.335 in MTB −4 dB. Bonferroni-corrected pairwise comparisons confirmed that MTB −4 dB had a higher boundary-error probability than each of the other listening conditions. No other pairwise comparisons were significant.

#### 3.2.3. Net Flow in the Directed Confusion Network

**Figure 5:**
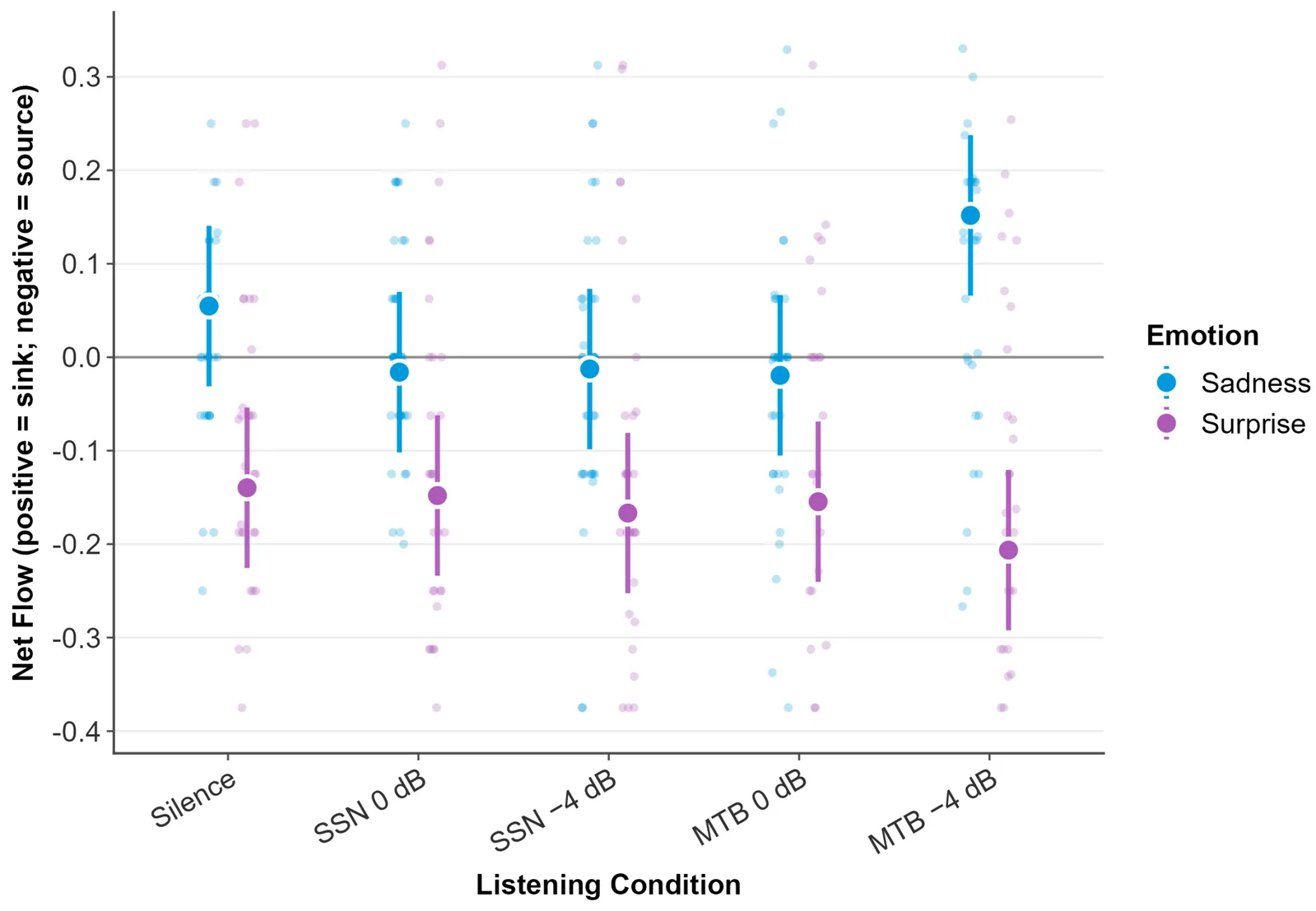
Net Flow for sadness and surprise across listening conditions. Light points show participant-level Net Flow values. Large points represent estimated marginal means from the focal Net Flow LMM, and error bars represent 95% confidence intervals. Net Flow was calculated as in-strength minus out-strength; positive values indicate a sink-like role, whereas negative values indicate a source-like role. SSN = speech-shaped noise; MTB = multi-talker babble; LMM = linear mixed-effects model.

Net Flow was calculated from the directed confusion matrices to summarize directional asymmetry, with Model 3 focusing on the a priori sadness and surprise nodes. Type III Wald χ^2^ tests showed no significant main effect of listening condition (χ^2^(4) = 1.633, *p* = 0.803). The main effect of focal emotion node was significant (χ^2^(1) = 56.631, *p* < 0.001), as was the listening condition × focal emotion node interaction (χ^2^(4) = 10.697, *p* = 0.030; Table 2). This interaction indicated that the Net Flow difference between sadness and surprise varied across listening conditions. Planned contrasts for Model 3 showed no significant listening-condition effects on Net Flow averaged across focal nodes, whereas the SNR contrast interacted with focal emotion node (*p* = 0.035). Model-estimated marginal means from Model 3 showed that surprise had negative Net Flow across listening conditions, indicating a source-like node pattern. Sadness Net Flow was near zero in most conditions but was positive in MTB −4 dB, indicating a more sink-like node pattern in this condition (Figure 5). In MTB −4 dB, estimated Net Flow was −0.206 for surprise and 0.152 for sadness. Bonferroni-corrected comparisons showed that sadness Net Flow in MTB −4 dB was higher than sadness Net Flow in SSN 0 dB, SSN −4 dB, and MTB 0 dB, whereas listening-condition differences for surprise were not significant.

As a robustness check, a four-node Net Flow sensitivity analysis was conducted using Model 4 to assess whether this focal sadness–surprise pattern was consistent with the broader directed confusion network. Type III Wald χ^2^ tests for Model 4 showed no significant main effect of listening condition (χ^2^(4) < 0.001, *p* = 1.000), but a significant effect of emotion node (χ^2^(3) = 278.828, *p* < 0.001) and a significant listening condition × emotion node interaction (χ^2^(12) = 23.328, *p* = 0.025; Table 2). Across listening conditions, happiness showed positive Net Flow, whereas anger and surprise showed negative Net Flow (Table S4). This four-node pattern was consistent with the focal Net Flow results, including negative Net Flow for surprise and positive Net Flow for sadness in MTB −4 dB.

## 4. Discussion

The present study showed that background masking affected Mandarin emotional prosody perception at both accuracy and confusion-structure levels. Recognition accuracy declined under masking, particularly under MTB at −4 dB SNR. Across emotions, estimated recognition accuracy was lowest for surprise and comparatively higher for sadness. Beyond accuracy, MTB at −4 dB increased sadness–high-arousal boundary errors, and Net Flow summarized node-specific directional asymmetries in the same confusion structure. These findings suggest that background masking affects emotional prosody perception not only by reducing overall recognition performance but also by modifying specific patterns of emotion-category confusions.

### 4.1. Recognition Accuracy Across Listening Conditions and Emotion Categories

The accuracy results showed a clear masking cost relative to silence. Consistent with H1, this finding suggests that emotional prosody perception is sensitive to acoustic degradation, even when semantic cues to emotion are reduced. Because emotional prosody is decoded from multiple vocal cues, including F0, intensity, duration, speech rate, and voice quality, masking may reduce access to these cues or make them less reliable (Banse & Scherer, 1996; Juslin & Laukka, 2003). This interpretation is consistent with broader speech-in-noise research showing that degraded auditory input reduces audibility and increases perceptual difficulty (Mattys et al., 2012). Thus, the masking-related decline indicates that vocal emotion decoding depends not only on the intended vocal expression, but also on the acoustic context in which it is heard.

Within the masked conditions, masker type further affected recognition accuracy. Accuracy was lower in MTB than in SSN, supporting the prediction that speech-like competition would be more disruptive than relatively steady spectral masking. This result is consistent with the distinction between energetic and informational masking. SSN primarily overlaps with the long-term speech spectrum and reduces audibility, whereas MTB contains speech-like temporal fluctuations, periodicity, and competing speech structure. These properties may have made the target emotional signal harder to segregate from the background, although the present design cannot directly separate energetic from informational masking. Prior work has similarly shown that babble-like maskers can create additional interference through target–masker similarity, stream selection, and attention drawn to competing speech (Brungart, 2001; Durlach et al., 2003; Kidd et al., 2008; Rosen et al., 2013). Because the present study did not directly measure listening effort or working memory, the MTB effect should not be interpreted as direct evidence of cognitive overload. Instead, it indicates greater perceptual difficulty with a speech-like competing masker than with a relatively steady speech-shaped masker.

In addition to masker type, SNR shaped recognition accuracy. Performance was poorer at −4 dB than at 0 dB SNR, and this SNR-related decline was larger in MTB than in SSN. This result supports H1 by showing that masker severity and masker type jointly affected emotional prosody recognition. In Mandarin, this pattern is particularly informative because it shows that emotional prosody can be strongly affected by adverse listening conditions despite sharing F0-related resources with lexical tone. Previous work suggests that Mandarin lexical tone perception can remain relatively robust under SSN and MTB masking, although performance varies with SNR and, within babble, the number of competing talkers (Wang & Xu, 2020). By contrast, emotional prosody appears to be more vulnerable to babble-like competition, consistent with evidence that Mandarin lexical tone perception may be less disrupted than vocal emotion recognition under MTB masking (Zhang et al., 2023). The present results extend this line of work by showing that, for Mandarin emotional prosody, the performance cost of masking was greatest when speech-like competition was combined with an unfavorable SNR.

Because the listening condition × target emotion interaction did not reach conventional statistical significance in the 36-participant analysis, the condition-specific emotion patterns should be interpreted descriptively. These descriptive results suggested possible category-related differences: surprise showed lower estimated accuracy, particularly in the MTB −4 dB condition, whereas sadness showed comparatively higher accuracy. This descriptive pattern is compatible with the category-related expectations of H1 and with previous Mandarin evidence showing lower recognition for happiness and pleasant surprise than for sadness and anger (Liu & Pell, 2012), as well as evidence that Mandarin emotional prosody recognition can be shaped by background speech and listener-related factors (Kuang et al., 2024). It is also consistent with the view that vocal emotions differ in recognizability and are decoded from cue combinations rather than single acoustic parameters (Banse & Scherer, 1996; Bänziger & Scherer, 2005; Juslin & Laukka, 2003; Larrouy-Maestri et al., 2025; Scherer, 1986; Scherer & Bänziger, 2004).

At the same time, the lower estimated accuracy for surprise in the present task suggests a possible context-dependent qualification of prior evidence that high-arousal emotional speech can enhance speech intelligibility and emotion recognition in noise (Alexander & Llanos, 2025). Although surprise is a higher-arousal category, its lower estimated accuracy in MTB −4 dB suggests that arousal-related salience alone may not guarantee robust category recognition under speech-like competition. The frequent surprise-to-happiness confusion provides additional descriptive support for this interpretation by suggesting that MTB masking may reduce the perceptual distinctiveness between emotionally similar high-arousal categories. Because acoustic predictors were not directly analyzed, this cue-based account remains tentative and should be understood as a response-level interpretation rather than direct evidence about acoustic mechanisms.

### 4.2. Confusion Structure and Directionality

The accuracy results identified where emotional prosody recognition was most impaired, but they did not reveal how incorrect responses were organized. The boundary-error analysis addressed this issue by showing that MTB at −4 dB SNR produced the highest probability of sadness–high-arousal boundary errors. This result supports the structured-confusion component of H2. Importantly, the effect was not simply a general consequence of masking, because the overall noise-versus-silence contrast was not significant for boundary errors. Instead, boundary errors were specifically affected by masker type, SNR, and their interaction. This suggests that the MTB −4 dB condition changed not only recognition performance but also how errors were distributed across emotion categories.

This boundary-error pattern is informative because sadness represented the relatively lower-arousal category, whereas happiness, anger, and surprise represented relatively higher-arousal categories. Russell’s (1980) circumplex model describes affective concepts along valence and arousal dimensions, while behavioral evidence suggests that vocal emotion perception involves both category-like structure and graded relations among affective states (Cowen et al., 2019; Laukka, 2005). From this perspective, the increase in sadness–high-arousal boundary errors suggests that MTB at −4 dB SNR reduced the response-level separation between sadness and the relatively higher-arousal categories. This does not imply that arousal perception itself was directly impaired, because participants did not provide arousal ratings. Rather, the result indicates that responses shifted across an arousal-relevant category boundary, revealing a change in confusion structure not captured by accuracy alone.

Whereas the boundary-error analysis described where errors were concentrated, the Net Flow analysis further revealed directional asymmetries in target–response confusions. Surprise showed a source-like pattern across listening conditions, whereas sadness showed a more sink-like pattern specifically in the MTB −4 dB condition. This finding provides qualified support for the directionality component of H2. The qualification reflects the nonsignificant main effect of listening condition on Net Flow and the nonsignificant condition-wise changes for surprise. Thus, masking did not produce a uniform shift in directed confusion patterns. Instead, the results point to a node-specific pattern in which surprise tended to be misidentified as other categories, whereas sadness attracted more misidentifications from other target emotions in the MTB −4 dB condition. The Net Flow findings therefore refined, rather than fully confirmed, H2 and should be understood as a summary of directional response asymmetry rather than independent node-level processes.

The source-like pattern for surprise aligns with its low recognition accuracy and frequent surprise-to-happiness confusions, suggesting that surprise was less stable as a target category and was positioned close to happiness in the observed response space. The sink-like pattern for sadness reflected a different form of asymmetry: sadness remained highly recognizable as a target, yet also attracted misidentifications from other emotions in the MTB −4 dB condition. This suggests that target robustness and response attraction can coexist. One possible explanation is that sadness-related vocal patterns remained comparatively accessible under the most adverse masking condition. Larrouy-Maestri et al. (2025) emphasized that emotional prosody is conveyed by combinations of acoustic cues rather than single category-specific parameters, and summarized prior evidence linking sadness to relatively low-arousal vocal characteristics, including a quiet and thin voice. From this cue-combination perspective, sadness may have remained accessible as a target while also becoming a plausible response when cues to other emotions were degraded under MTB −4 dB. However, because the present study did not directly analyze acoustic predictors, this interpretation should be considered a possible explanation of the observed behavioral pattern rather than evidence for specific acoustic mechanisms.

This directional-confusion pattern is consistent with network-based approaches in cognitive science, which use relational measures to describe links among categories or concepts (Baronchelli et al., 2013; Siew et al., 2019). Applied to emotional prosody, a network-based approach can help summarize asymmetries in target–response confusions. The four-node sensitivity analysis yielded a similar node-dependent pattern, providing a robustness check for the focal sadness–surprise contrast. However, Net Flow should be interpreted as a behavioral index of directed confusion asymmetry, not as evidence for a fixed underlying emotion network or invariant node roles across contexts.

### 4.3. Implications for Emotional Prosody Perception Under Masking

Taken together, the accuracy and confusion findings indicate that masking influences not only performance outcomes but also the structure of perceptual errors. This distinction extends the view that vocal emotion perception involves both discrete categories and graded relations among emotions (Cowen et al., 2019): accuracy indexed whether listeners selected the intended category, whereas confusion analyses revealed whether errors were diffuse or structured, which category boundaries were crossed, and which response categories attracted misidentifications.

This interpretation is consistent with accounts of vocal emotion communication in which speakers express affective states through probabilistic acoustic cues and listeners use these cues to infer emotion-relevant meaning (Juslin & Laukka, 2003; Scherer, 2003). The present results indicate that this decoding process is shaped by listening conditions: when background speech competes with the target utterance, response errors may become systematically reorganized rather than merely more frequent. Thus, background masking, especially speech-like competition, may influence listeners’ emotion judgments by changing emotion-confusion patterns as well as reducing recognition accuracy.

Within the present young-adult Mandarin-speaking sample, these results suggest that emotional-prosody errors under masking involved predictable response tendencies rather than random guessing. In the present task, this was reflected in frequent surprise-to-happiness confusions and greater sadness-directed responses under severe MTB masking. Such patterns are important because emotional prosody contributes to judgments of urgency, intention, interpersonal stance, and affective meaning. However, whether similar or stronger structured-error patterns occur in older adults, listeners with hearing loss, or other potentially vulnerable groups remains an empirical question for future research.

### 4.4. Limitations and Future Directions

#### 4.4.1. Limitations

These findings should be interpreted in light of several limitations related to the participant sample, experimental design, stimulus characteristics, and measurement approach. First, participant characteristics and hearing assessment should be considered. Hearing status was assessed by self-report rather than by audiometric screening: participants reported no known hearing impairment, but no objective hearing test was conducted. This limitation should be considered when interpreting performance under the more demanding masking conditions, especially MTB at −4 dB SNR. In addition, the sample consisted of young adult Mandarin speakers, some of whom reported regional-variety backgrounds. Systematic information about non-Chinese language experience was not collected, and the sample size was not sufficient to model these background variables as predictors.

Second, several aspects of the experimental design and stimulus construction limit the generality of the findings. Although block order was counterbalanced and block position was statistically controlled in the accuracy model, the blocked presentation cannot fully disentangle listening-condition effects from residual adaptation, fatigue, or practice effects. In addition, because only four emotion categories were included, the sadness–high-arousal boundary should be interpreted as a design-based contrast rather than a complete model of valence–arousal organization. Stimulus construction also imposes constraints on interpretation: although emotionally neutral utterances were used to construct the MTB and were separated from the target corpus, the masker utterances were not systematically matched to the target sentences in topic or prosodic style. Naturally produced neutral speech may therefore still contain residual prosodic variation that cannot be completely controlled. Finally, the study did not directly model acoustic predictors, analyze response times as outcome measures, or include independent listening-effort measures.

#### 4.4.2. Future Directions

These limitations identify several directions for future research. Future studies should combine objective hearing assessment with broader participant samples, including older adults and listeners with hearing loss, to determine whether the observed confusion patterns generalize to populations more vulnerable to adverse listening conditions. Future studies using larger emotion sets, more naturalistic speech materials, and alternative presentation designs, together with acoustic cue modeling, would further clarify the mechanisms underlying emotion-category differences under masking.

Taken together, the present study shows that confusion-based measures extend accuracy-based accounts of emotional prosody perception under masking. By identifying boundary-crossing errors and directional response tendencies, these measures help characterize how degraded listening conditions alter emotion judgments.

## 5. Conclusions

This study provides evidence that background masking affects Mandarin emotional prosody perception by reducing recognition accuracy and altering the organization of response errors. The findings lead to three main conclusions. First, recognition accuracy declined under masking, with the greatest impairment occurring in the MTB −4 dB condition, where speech-like competition was presented at the most adverse SNR. Second, estimated recognition accuracy differed across emotion categories, with lower accuracy for surprise and relatively robust performance for sadness. Third, confusion analyses revealed structured errors beyond overall accuracy, with MTB −4 dB associated with increased sadness–high-arousal boundary errors and node-specific directional asymmetries. Within this directed structure, surprise generally showed a source-like pattern, whereas sadness showed a more sink-like pattern under MTB −4 dB. Together, these findings indicate that severe adverse listening conditions do not merely reduce category identification but can also affect how emotional prosody errors are organized and directed.

## Figures and Tables

**Table 1:** Fixed-effect tests and planned condition contrasts from Model 1.

Part A. Type III Wald tests					
Effect			χ^2^	df	*p*
Listening condition			71.250	4	<0.001
Emotion			49.403	3	<0.001
Block			40.533	1	<0.001
Listening condition × emotion			21.008	12	0.050
Part B. Planned condition contrasts					
Contrast	Estimate	*SE*	*OR*	95% CI for *OR*	*p*
Noise vs. silence	−0.115	0.022	0.891	[0.854, 0.930]	<0.001
MTB vs. SSN	−0.162	0.052	0.850	[0.768, 0.942]	0.002
0 dB vs. −4 dB SNR	0.223	0.045	1.249	[1.145, 1.363]	<0.001
Masker type × SNR	0.107	0.046	1.113	[1.017, 1.217]	0.020

Note. Accuracy was analyzed with a trial-level binomial GLMM using a logit link. Part A reports Type III Wald χ^2^ tests; Part B reports fixed-effect coefficients for planned orthogonal condition contrasts from the same model. Estimates are on the log-odds scale, and ORs are odds ratios. Listening condition levels were ordered as silence, SSN 0 dB, SSN −4 dB, MTB 0 dB, and MTB −4 dB; contrast coding followed this order and is described in Section 2.4.2. Block position was included as a centered covariate. GLMM = generalized linear mixed-effects model; OR = odds ratio; SSN = speech-shaped noise; MTB = multi-talker babble; SNR = signal-to-noise ratio.

**Table 2:** Type III Wald tests for the boundary-error and Net Flow models.

Model	Analysis	Effect	χ^2^	df	*p*
Model 2	Boundary-error GLMM	Listening condition	40.602	4	<0.001
Model 3	Focal Net Flow LMM	Listening condition	1.633	4	0.803
Model 3	Focal Net Flow LMM	Focal emotion node	56.631	1	<0.001
Model 3	Focal Net Flow LMM	Listening condition × focal emotion node	10.697	4	0.030
Model 4	Four-node Net Flow sensitivity LMM	Listening condition	0.000	4	1.000
Model 4	Four-node Net Flow sensitivity LMM	Emotion node	278.828	3	<0.001
Model 4	Four-node Net Flow sensitivity LMM	Listening condition × emotion node	23.328	12	0.025

Model 2 was a binomial GLMM predicting the probability that an included error crossed the sadness–high-arousal boundary. Model 3 was the primary focal Net Flow LMM and included sadness and surprise as focal nodes. Model 4 was a complementary four-node Net Flow sensitivity model including all emotion nodes. Model 4 was interpreted as a robustness check because Net Flow values sum to zero within each participant-by-condition network. GLMM = generalized linear mixed-effects model; LMM = linear mixed-effects model.

## Data Availability

The participant-level trial data are available from the corresponding author upon reasonable request due to restrictions in the informed consent, which did not include permission for public sharing of individual-level behavioral data. Restrictions apply to the availability of speech stimuli and masker materials because they were derived from third-party speech databases subject to licensing terms. Analysis scripts and group-level summary data are available from the corresponding author upon reasonable request.

## Supplementary Materials

The following supporting information can be downloaded at: https://www.mdpi.com/article/10.3390/bs16091605/s1. Table S1: Descriptive acoustic characteristics of clean target tokens by emotion category; Figure S1: Spectral match between the target speech long-term average spectrum and the generated speech-shaped noise; Table S2: Estimated marginal means for recognition accuracy; Table S3: Row-normalized target–response confusion matrices; Table S4: Four-node Net Flow estimates across listening conditions.
