# Supplementary material for: Background Masking Alters Accuracy and Confusion Structure in Mandarin Emotional Prosody Perception
Source: Behav Sci (Basel). 2026 Sep 9;16(9):1605. doi: 10.3390/bs16091605 (PMC13603255; doi:10.3390/bs16091605)
Supplement: Supplementary file 1 [file behavsci-16-01605-s001.zip › behavsci-4494358-supplementary.pdf]

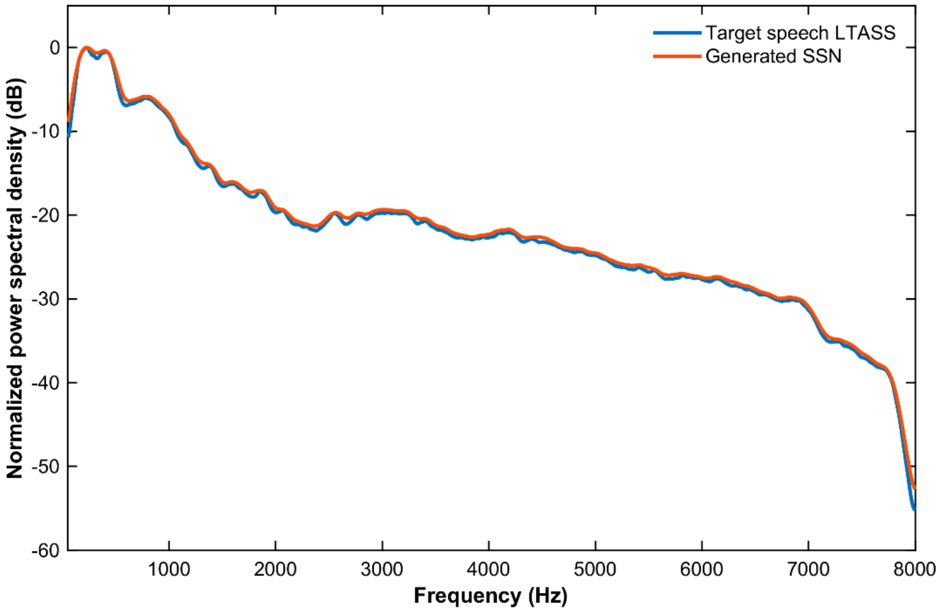

Figure S1. Spectral match between the target speech long-term average spectrum (LTASS) and the generated speech-shaped noise (SSN). The long-term average spectrum of the concatenated target speech tokens was compared with the spectrum of the 60-s speech-shaped noise template. Spectra were estimated using Welch's method with a 2048-sample Hamming window, 50% overlap, and a 4096-point fast Fourier transform, and were then smoothed using an approximately 100-Hz moving average. Spectra were normalized for shape comparison. The Pearson correlation between the smoothed normalized spectra over 50–8,000 Hz was $r > 0.99$.

Table S1. Descriptive Acoustic Characteristics of Clean Target Tokens by Emotion Category

Emotion	Tokens (N)	Duration (s)	Mean F0 (Hz)	Median F0 (Hz)	F0 SD (Hz)	F0 range (Hz)
Happiness	16	1.532 ± 0.285	237.78 ± 83.21	244.09 ± 89.77	62.60 ± 26.71	194.53 ± 79.30
Sadness	16	1.856 ± 0.487	179.30 ± 57.12	176.89 ± 59.31	33.31 ± 14.66	107.90 ± 52.38
Anger	16	1.240 ± 0.177	228.40 ± 71.11	228.43 ± 76.94	51.01 ± 20.76	161.30 ± 61.93
Surprise	16	1.271 ± 0.179	265.16 ± 80.35	267.95 ± 92.33	68.29 ± 25.41	205.42 ± 64.57

Note. Values are M ± SD across tokens. Acoustic measures were calculated from the clean target tokens before masker mixing and before the addition of 300-ms leading and trailing silences. Duration refers to the effective speech portion. F0 was extracted using Praat with sex-specific pitch ranges, and F0 statistics were calculated over voiced frames only. F0 range refers to the difference between the 95th and 5th percentiles of voiced-frame F0 values. RMS levels are not shown because all target tokens were RMS-normalized before experimental mixing. These acoustic summaries are descriptive and were not entered as predictors in the behavioral response models.

Table S2. Estimated Recognition Accuracy by Listening Condition and Target Emotion

Listening condition	Target emotion	Estimated accuracy	SE	95% CI
Silence	Happiness	0.854	0.041	[0.755, 0.917]
Silence	Sadness	0.980	0.008	[0.958, 0.990]
Silence	Anger	0.927	0.022	[0.869, 0.960]
Silence	Surprise	0.700	0.066	[0.558, 0.811]
SSN 0 dB	Happiness	0.859	0.039	[0.763, 0.921]
SSN 0 dB	Sadness	0.958	0.014	[0.919, 0.978]
SSN 0 dB	Anger	0.903	0.028	[0.832, 0.946]
SSN 0 dB	Surprise	0.661	0.070	[0.515, 0.782]
SSN −4 dB	Happiness	0.859	0.040	[0.763, 0.921]
SSN −4 dB	Sadness	0.954	0.016	[0.912, 0.976]
SSN −4 dB	Anger	0.832	0.044	[0.728, 0.902]
SSN −4 dB	Surprise	0.615	0.074	[0.465, 0.746]
MTB 0 dB	Happiness	0.871	0.037	[0.780, 0.928]
MTB 0 dB	Sadness	0.945	0.018	[0.898, 0.972]
MTB 0 dB	Anger	0.878	0.034	[0.794, 0.930]
MTB 0 dB	Surprise	0.659	0.070	[0.512, 0.780]
MTB −4 dB	Happiness	0.759	0.059	[0.627, 0.856]
MTB −4 dB	Sadness	0.927	0.023	[0.868, 0.961]
MTB −4 dB	Anger	0.737	0.060	[0.606, 0.837]
MTB −4 dB	Surprise	0.505	0.078	[0.357, 0.652]

Note. Values are estimated marginal means from the trial-level accuracy GLMM and are presented on the response-probability scale. Confidence intervals were back-transformed from the logit scale. GLMM = generalized linear mixed-effects model; CI = confidence interval; SSN = speech-shaped noise; MTB = multi-talker babble.

Table S3. Complete Directed Edge Probabilities in the Target–Response Confusion Networks

Listening condition	Target emotion	Response emotion	n	target N	Probability
Silence	Happiness	Sadness	33	569	0.058
Silence	Happiness	Anger	6	569	0.011
Silence	Happiness	Surprise	74	569	0.130
Silence	Sadness	Happiness	21	572	0.037
Silence	Sadness	Anger	3	572	0.005
Silence	Sadness	Surprise	11	572	0.019
Silence	Anger	Happiness	39	574	0.068
Silence	Anger	Sadness	6	574	0.010
Silence	Anger	Surprise	30	574	0.052
Silence	Surprise	Happiness	153	573	0.267
Silence	Surprise	Sadness	28	573	0.049
Silence	Surprise	Anger	15	573	0.026
SSN 0 dB	Happiness	Sadness	19	570	0.033
SSN 0 dB	Happiness	Anger	5	570	0.009
SSN 0 dB	Happiness	Surprise	90	570	0.158
SSN 0 dB	Sadness	Happiness	41	573	0.072
SSN 0 dB	Sadness	Anger	10	573	0.017
SSN 0 dB	Sadness	Surprise	9	573	0.016
SSN 0 dB	Anger	Happiness	56	576	0.097
SSN 0 dB	Anger	Sadness	7	576	0.012
SSN 0 dB	Anger	Surprise	32	576	0.056
SSN 0 dB	Surprise	Happiness	167	574	0.291
SSN 0 dB	Surprise	Sadness	25	574	0.044
SSN 0 dB	Surprise	Anger	24	574	0.042
SSN −4 dB	Happiness	Sadness	36	573	0.063
SSN −4 dB	Happiness	Anger	5	573	0.009

**Listening condition**	**Target emotion**	**Response emotion**	**n**	**target N**	**Probability**
SSN −4 dB	Happiness	Surprise	75	573	0.131
SSN −4 dB	Sadness	Happiness	46	570	0.081
SSN −4 dB	Sadness	Anger	9	570	0.016
SSN −4 dB	Sadness	Surprise	11	570	0.019
SSN −4 dB	Anger	Happiness	72	572	0.126
SSN −4 dB	Anger	Sadness	8	572	0.014
SSN −4 dB	Anger	Surprise	54	572	0.094
SSN −4 dB	Surprise	Happiness	186	570	0.326
SSN −4 dB	Surprise	Sadness	15	570	0.026
SSN −4 dB	Surprise	Anger	33	570	0.058
MTB 0 dB	Happiness	Sadness	32	570	0.056
MTB 0 dB	Happiness	Anger	9	570	0.016
MTB 0 dB	Happiness	Surprise	67	570	0.118
MTB 0 dB	Sadness	Happiness	52	572	0.091
MTB 0 dB	Sadness	Anger	8	572	0.014
MTB 0 dB	Sadness	Surprise	10	572	0.017
MTB 0 dB	Anger	Happiness	54	572	0.094
MTB 0 dB	Anger	Sadness	3	572	0.005
MTB 0 dB	Anger	Surprise	49	572	0.086
MTB 0 dB	Surprise	Happiness	166	570	0.291
MTB 0 dB	Surprise	Sadness	24	570	0.042
MTB 0 dB	Surprise	Anger	24	570	0.042
MTB −4 dB	Happiness	Sadness	62	568	0.109
MTB −4 dB	Happiness	Anger	19	568	0.033
MTB −4 dB	Happiness	Surprise	85	568	0.150
MTB −4 dB	Sadness	Happiness	61	565	0.108
MTB −4 dB	Sadness	Anger	7	565	0.012
MTB −4 dB	Sadness	Surprise	16	565	0.028

**Listening condition**	**Target emotion**	**Response emotion**	**n**	**target N**	**Probability**
MTB −4 dB	Anger	Happiness	86	571	0.151
MTB −4 dB	Anger	Sadness	32	571	0.056
MTB −4 dB	Anger	Surprise	61	571	0.107
MTB −4 dB	Surprise	Happiness	171	570	0.300
MTB −4 dB	Surprise	Sadness	78	570	0.137
MTB −4 dB	Surprise	Anger	33	570	0.058

Note. Each row represents an off-diagonal target-to-response confusion within a listening condition. n is the number of trials for the listed target–response pair, and Target N is the total number of valid trials for that target emotion in that listening condition. Edge probability was calculated as n / Target N and represents the row-normalized conditional probability of the response emotion given the target emotion. Diagonal responses are omitted because they represent correct recognition. SSN = speech-shaped noise; MTB = multi-talker babble.

Table S4. Four-node Net Flow Sensitivity Analysis

Panel A. Four-node Net Flow Estimated Marginal Means Averaged across Listening Conditions

Emotion	Estimated Net Flow	SE	95% CI	p
Happiness	0.264	0.020	[0.224, 0.304]	< 0.001
Sadness	0.032	0.020	[−0.008, 0.072]	0.119
Anger	−0.132	0.020	[−0.172, −0.093]	< 0.001
Surprise	−0.163	0.020	[−0.203, −0.123]	< 0.001

Panel B. Four-node Net Flow Estimated Marginal Means by Listening Condition and Emotion

Listening condition	Emotion	Estimated Net Flow	*SE*	*95% CI*	*p*
Silence	Happiness	0.173	0.045	[0.084, 0.263]	< 0.001
Silence	Sadness	0.055	0.045	[−0.034, 0.144]	0.228
Silence	Anger	−0.089	0.045	[−0.178, 0.001]	0.051
Silence	Surprise	−0.140	0.045	[−0.229, −0.051]	0.002
SSN 0 dB	Happiness	0.261	0.045	[0.172, 0.350]	< 0.001
SSN 0 dB	Sadness	−0.016	0.045	[−0.105, 0.073]	0.725
SSN 0 dB	Anger	−0.097	0.045	[−0.186, −0.008]	0.033
SSN 0 dB	Surprise	−0.148	0.045	[−0.237, −0.059]	0.001
SSN −4 dB	Happiness	0.332	0.045	[0.243, 0.421]	< 0.001
SSN −4 dB	Sadness	−0.013	0.045	[−0.102, 0.076]	0.781
SSN −4 dB	Anger	−0.153	0.045	[−0.242, −0.064]	0.001
SSN −4 dB	Surprise	−0.167	0.045	[−0.256, −0.078]	< 0.001
MTB 0 dB	Happiness	0.287	0.045	[0.198, 0.376]	< 0.001
MTB 0 dB	Sadness	−0.019	0.045	[−0.108, 0.070]	0.668
MTB 0 dB	Anger	−0.113	0.045	[−0.202, −0.024]	0.013
MTB 0 dB	Surprise	−0.155	0.045	[−0.244, −0.066]	0.001

Listening condition	Emotion	Estimated Net Flow	*SE*	*95% CI*	*p*
MTB −4 dB	Happiness	0.265	0.045	[0.176, 0.354]	< 0.001
MTB −4 dB	Sadness	0.152	0.045	[0.063, 0.241]	0.001
MTB −4 dB	Anger	−0.210	0.045	[−0.299, −0.121]	< 0.001
MTB −4 dB	Surprise	−0.206	0.045	[−0.295, −0.117]	< 0.001

Note. Net Flow was calculated as in-strength minus out-strength. Positive values indicate a sink-like role, whereas negative values indicate a source-like role. The four-node sensitivity analysis included happiness, sadness, anger, and surprise. Because Net Flow values across all nodes within each participant-by-condition network sum to zero, this analysis was interpreted as a robustness check. CI = confidence interval; SSN = speech-shaped noise; MTB = multi-talker babble.
